# Isolation, characterization and antimicrobial evaluation of a novel compound N-octacosan 7*β* ol, from *Fumaria parviflora* Lam

**DOI:** 10.1186/1472-6882-14-98

**Published:** 2014-03-12

**Authors:** Mohammad Jameel, Mohammad Islamuddin, Abuzer Ali, Farhat Afrin, Mohammed Ali

**Affiliations:** 1 Phytochemistry research Lab., Department of Pharmacognosy and Phytochemistry, Faculty of Pharmacy, Jamia Hamdard, New Delhi 110062, India; 2 Parasite Immunology Lab., Department of Biotechnology, Faculty of Science, Jamia Hamdard, New Delhi 110062, India

**Keywords:** *Fumaria parviflora*, Homaira, Shahtrah, Leishmanial potential, Novel, Isolation

## Abstract

**Background:**

*Fumaria parviflora* Lam. (Fumaraceae) is widely used in traditional as well as folkloric system of medicine from ancient. It is commonly known as ‘Pitpapra’ or ‘Shahtrah’ in Indian traditional system of medicine and used for treating numerous ailments like diarrhea, fever, influenza, blood purifier and other complications. The object of the present study was to evaluate the Antileishmanial, antibacterial, antifungal and cytotoxic potential of isolated compound.

**Methods:**

Methanolic extract of whole plant of *Fumaria parviflora* was dried under reduced pressure to obtain a dark brown residue which was adsorbed on silica gel column grade (60–120 mesh) to obtain a slurry and chromatographed over silica gel loaded column in petroleum ether – chloroform (3:1, 1:1 and 1:3 *v/v*). The *in vitro* antileishmanial evaluation of isolated compound against *Leishmania donovani* promastigotes was investigated by growth kinetics assay, reversibility assay, analysis of cellular morphology, adverse toxicity and determination of 50% growth inhibitory concentration (GI_50_). Disc diffusion and broth micro dilution methods were used to study the antibacterial (Gram + *Staphylococcus epidermidis* and *Bacillus subtilis; Gram - Escherichia coli* and *Salmonella typhimurium*) and antifungal (*Candida albicans* and *Aspergillus niger*) potential *in vitro.*

**Results:**

Structure elucidation by spectral data analysis revealed a novel compound, n-octacosan-7β-ol (**OC**), yield (0.471%), having significant antimicrobial activity against *Leishmania donovani* promastigotes, *Staphylococcus epidermidis, Escherichia coli, Candida albicans* and *Aspergillus niger in vitro* with GI_50_ = 5.35, MIC 250, MIC 250 and MFC 500 and MIC 250 μg ml^-1^ respectively. The isolated compound did not show adverse effect against mammalian macrophages.

**Conclusions:**

The available evidence of compound suggested that it may be used as antimicrobial agent in future and may provide new platform for drug discovery programmes for leishmaniasis.

## Background

The traditional medicine has been a vibrant, innovation-driven highly successful component of global industry and confluence of spectacular advances in chemistry, molecular biology, genomics and the cognate fields of spectroscopy, chromatography and crystallography led to the new findings and development of numerous novel curative agents for the treatment of a wide spectrum of ailments [1]. They have formed the basis of sophisticated traditional medicine systems among which Ayurvedic, Unani and Chinese systems have given ascent to crucial unmatched lead molecules still in use today [2]. The number of higher medicinal plant species on this planet is estimated at 250,000 [3] but of these only about 6% have been screened for biological activity, 15% have been evaluated phytochemically [4] and only about 0.75% herbal drugs have been studied in clinical trials [5]. So the search for new molecules nowadays has taken a faintly different route where the science of ethanomedicine is being used as a guide to lead the chemist towards different sources and classes of molecules [2].

*Fumaria parviflora* Lam. is widely used in traditional and as well as folkloric system of medicine named as “fumitory, earth smoke, beggary, fumus, vapor and fumitory or wax dolls” in English [6]. It is locally known as ‘Pitpapra’ or ‘Shahtrah’ in India [7], “Homaira” in Saudi Arabia [8]. Phytochemical investigation of this medicinal plant revealed the presence of several alkaloids [9,10], and shown to possess pharmacological activities like antipyretic [11], hepatoprotective [12]*,* hypoglycemic [13], antidiarrheal, antispasmodic, bronchodilator [14], anthelmintic [15], antieczema [16] and nematocidal activities [17].

Visceral leishmaniasis is an infectious disease caused by protozoan parasite, *leishmania donovani* which is considered as a major public health trouble in developing countries [18]. Primary health care systems are not always significant to use the herbal medicines in common practice because very few studies were carried out to investigate the potential use of these medicinal plants in treatment of parasitic diseases [19]. The chemotherapeutic interventions against visceral leishmaniasis (VL) are limited and facing severe concerns of toxicity, high cost, and drug resistance [20]. In view of that concern an integrated approach towards the discovery and development of novel chemical entity with ethanopharmacological implications for promotion and development of leads for *leishmaniasis* as well as traditional medicine, remains a significant hope in the current, target-rich scenario which may offer unprecedented diversity in structures and bioactivity. So isolation, characterization and antimicrobial evaluation has been aimed for novel bioactive compound by chromatographic techniques, and bacterial strain (Gram + *Staphylococcus epidermidis* and *Bacillus subtilis; Gram - Escherichia coli* and *Salmonella typhimurium*)*,* fungal strain (*Candida albicans* and *Aspergillus niger*) and *Leishmania donovani* promastigotes.

## Methods

### Materials

M199 medium, RPMI 1640 medium were obtained from Sigma-Aldrich, fetal bovine serum (FBS) from Gibco- BRL, DMSO from SRL and methanol from Merck. All other chemicals were from Sigma-Aldrich unless otherwise stated. Melting point was determined on a Perfit apparatus without correction. The IR spectrum was measured in KBr pellet on a Bio-Red FT-IR spectrometer. UV spectrum was obtained in methanol with a Lambda Bio 20 spectrometer, ^1^H (400 MHz), ^13^C (100 MHz), NMR spectra were recorded on Bruker spectrospin spectrometer. CDCl_3_ (sigma-Aldrich) were used as solvent and TMS as an internal standard. ESI- MS analysis was performed on a Synapt Mass spectrometer (Waters) equipped with direct inlet probe system. Column chromatography separations were carried out on column grade silica gel (Merck, 60–120 mesh). Precoated silica gel plates (Merck, Silica gel 60 F_254_) were used for analytical thin layer chromatography (TLC) visualized by exposure to iodine and UV radiations. Microbial strains used for the antimicrobial evaluation, a panel which included laboratory control bacterial strains (Gram + *Staphylococcus epidermidis* and *Bacillus subtilis; Gram - Escherichia coli* and *Salmonella typhimurium*) fungal strains (*Candida albicans* and *Aspergillus niger*), and *Leishmania donovani* promastigote. Streptomycin, amphotericin B and DMSO analytical grade used for positive and negative control respectively.

### Methods

#### Plant material

The *Fumaria parviflora* Lam. (whole plant) was collected from the herbal garden of Jamia Hamdard, New Delhi and identified by Prof. Javed Ahmad, Incharge herbal garden. A specimen voucher of the drug was deposited to the Phytochemistry Research Lab (PRL) with a reference number PRL-JH/2011/05.

#### Parasite culture

*L. donovani* strain AG83 was a kind gift from Dr. Nahid Ali (IICB, Kolkata, India) and was maintained *in vivo* in BALB/c mice. Promastigotes were maintained in medium M199 supplemented with 100 U ml^-1^sodium penicillin G and 100 μg ml streptomycin^1^ sulfate (incomplete medium) and 10% heat-inactivated FBS at 22°C, and subcultured every 72 h in the same medium at a mean density of 2 × 10^6^ cells/ml [21].

#### Preparation of crude extract and isolation

The dried *F. parviflora* Lam. whole plant (1.25 kg) was coarsely powdered and extracted with methanol for 72 h using a Soxhlet extractor. The extract was dried under reduced pressure to obtain a dark brown residue (190 g). The residue (100 g) was dissolved in minimum amount of methanol and adsorbed on silica gel column grade (60–120 mesh) to obtain slurry. The slurry was dried in air and chromatographed over silica gel column loaded in petroleum ether. The column was eluted with petroleum ether and petroleum ether –chloroform (3:1, 1:1, 1:3 *v/v*) mixtures.

### General procedure for antimicrobial evaluation

#### Antimicrobial activity

Agar disc diffusion method and broth microdilution method were employed for the determination of antibacterial and antifungal activities. The minimum inhibitory concentration (MIC), minimum bactericidal (MBC), minimum fungicidal concentration (MFC) [22] and the inhibition zone of the OC against the test microorganisms were determined by the broth microdilution method. The MIC, MBC, MFC and the inhibition zone of the *n*-octacosan-7β-ol (**OC**) were also determined in parallel experiments in order to control the sensitivity of the test microorganisms.

A suspension of the bacterial strain (Gram+*Staphylococcus epidermidis* and *Bacillus subtilis; Gram - Escherichia coli* and *Salmonella typhimurium*)*,* fungal strain (*Candida albicans* and *Aspergillus niger*) (0.1 ml of 10^5^ CFU ml^-1^) was spread on the solid media plates. Nutrient agar and Czapek Dox Agar sterilized in a flask and cooled to 45~ 50°C were distributed to sterilized Petri dishes with a diameter of 9 cm (15 ml). The filter paper discs (6 mm in diameter) were individually impregnated with 50 μl (1000 μg ml^-1^) of the OC and then placed onto the agar plates which had previously been inoculated with the tested microorganisms. The plates were inoculated with bacterial strains incubated at 37°C for 24 h and at 28°C for 48 h for the fungal strains. The diameters were measured in millimeters. Streptomycin (10 μg/disk) was used as a positive control for bacteria, amphotericin B (5 μg/disk) as a positive control for fungi, and DMSO was used as the negative control. All assays were done in duplicate. A broth microdilution method was used to determine the MIC, MBC and MFC according to the National Committee for Clinical Laboratory Standards [23]. All tests were performed in Nutrient broth and Czapek-Dox broth supplemented with ethanol at final concentration of 0.5% (*v/v*) for all microorganisms. Two folds serial dilutions of the **OC** were prepared in a 96-well microtiter plate ranged from 1000 μg ml^-1^ to 1.038 μg ml^-1^. Overnight broth cultures of each strain were prepared and the final concentration in each well was adjusted to 10^5^ CFU ml^-1^for bacterial strains and fungal strains. 96-well microtiter plate injected with fungal strain was incubated at 28°C for 48 h, and the bacteria were incubated at 37°C for 24 h. The MIC is defined as the lowest concentration of the **OC** at which the microorganism does not demonstrate visible growth. To determine MBC and MFC, 10 μl broth was taken from each well and inoculated in Nutrient for 24 h at 37°C for bacteria or in Czapek Dox Agar for 48 h at 28°C for the fungi. The MBC is defined as the lowest concentration of the **OC** at which inoculated bacteria were completely killed. The MFC is defined as the lowest concentration of the **OC** at which inoculated fungi were completely killed. All determinations were performed in duplicate and two growth controls consisting of Nutrient Agar and Czapek Dox agar medium were included. The streptomycin and amphotericin B served as a positive control.

#### Growth kinetics assay

Promastigotes of *L. donovani* strain MHOM/IN/83/AG83 (2 × 10^6^ cells ml^-1^) were incubated in the presence of **OC** in M199 containing 10% FBS (complete medium) at a concentration of 100 μg ml^-1^. Pentamidine (100 μg ml^-1^) served as the reference antileishmanial drug, while 0.2% DMSO, which represented the highest concentration in the test compound, was used as a solvent control. Parasites in medium alone were taken as control. Viable parasites were enumerated for 7 days using a phase-contrast microscope under a 40X objective [24].

#### Growth reversibility assay

To confirm the leishmanicidal effect of **OC**, treated and untreated parasites after 7 days of incubation were washed twice with incomplete medium and finally resuspended in complete medium and cultured at 22°C for a further 96 h. The viability of the parasites was ascertained microscopically [24].

#### Analysis of cellular morphology

Morphology of the promastigotes was evaluated after 96 h of treatment with the test **OC**, pentamidine and or 0.2% DMSO and photomicrographs were taken at 400x magnification under phase-contrast microscope [25].

#### Determination of GI_50_

To determine the GI_50_ (concentration of compound that inhibited growth of parasites by 50%), promastigotes at a density of 2 × 10^6^ cells ml^-1^ were incubated in triplicate with or without **OC** at serial three fold dilutions starting from 100 μg ml^-1^ for 96 h. Pentamidine served as the reference drug [26].

#### Cytotoxicity to mammalian cells

Macrophages were collected by peritoneal lavage from starch-stimulated mice. Peritoneal cells were collected in RPMI 1640 medium (incomplete), pelleted by centrifugation at 800 x g for 10 min at 4°C, washed twice and finally resuspended in complete medium. Macrophages at a cell density of 1 × 10^6^ cells ml^-1^ were incubated with OC at varying concentrations for 48 h in a CO_2_ incubator (5% CO_2_, 37°C). Pentamidine served as reference drug and 0.2% DMSO as solvent control. Macrophages without any treatment were taken as control. Cells were observed under phase-contrast microscope and viability was ascertained after trypan blue staining [26].

## Results

### Structural elucidation of isolated compound

Elution of the column with ratio of petroleum ether-chloroform afforded star-shaped colorless of **OC**, recrystallized from chloroform-methanol (1:1), 3.1 g (0.471% yield); R_f_ 0.54 ± 0.02 (petroleum ether-chloroform, 1:1 *v/v*), m.p. 74–75°C; UV λ max (MeOH); 209 nm (log ϵ 3.6); IR λ max (KBr); 3318, 2954, 2848, 1465, 1378, 1236, 1134, 857, 721 cm^−1^; ^1^H NMR (CDCl_3_); δ 3.67 (1H, brm, w_1/2_ = 14.1 Hz_,_ H-7α), 2.36 (2H, m H_2_ -6), 2.14 (2H m, H_2_-8), 1.70 (4H, m, 2xCH_2_), 1.51 (8H, m, 4x CH_2_), 1.34 (34H, 17x CH_2_), 0.98 (3H, t, *J*= 6.0, Me-1), 0.95 (3H, t, *J*= 6.8, Me-28); ^13^C NMR (CDCl_3_); δ 72.06 (C-7), 37.50 (CH_2_), 31.95 (CH_2_), 31.91 (CH_2_), 29.73 (6x CH_2_), 29.67 (7x CH_2_), 29.61 (5x CH_2_), 29.40 (CH_2_), 29.36 (CH_2_), 25.68 (CH_2_), 22.72 (CH_2_), 14.16 (Me-1, Me-28). +ve ESI-MS *m/z* (rel. int.); 411 [M^+1^]^+^ (9.6), (C_28_H_59_O), 325(100).

**Figure 1:**
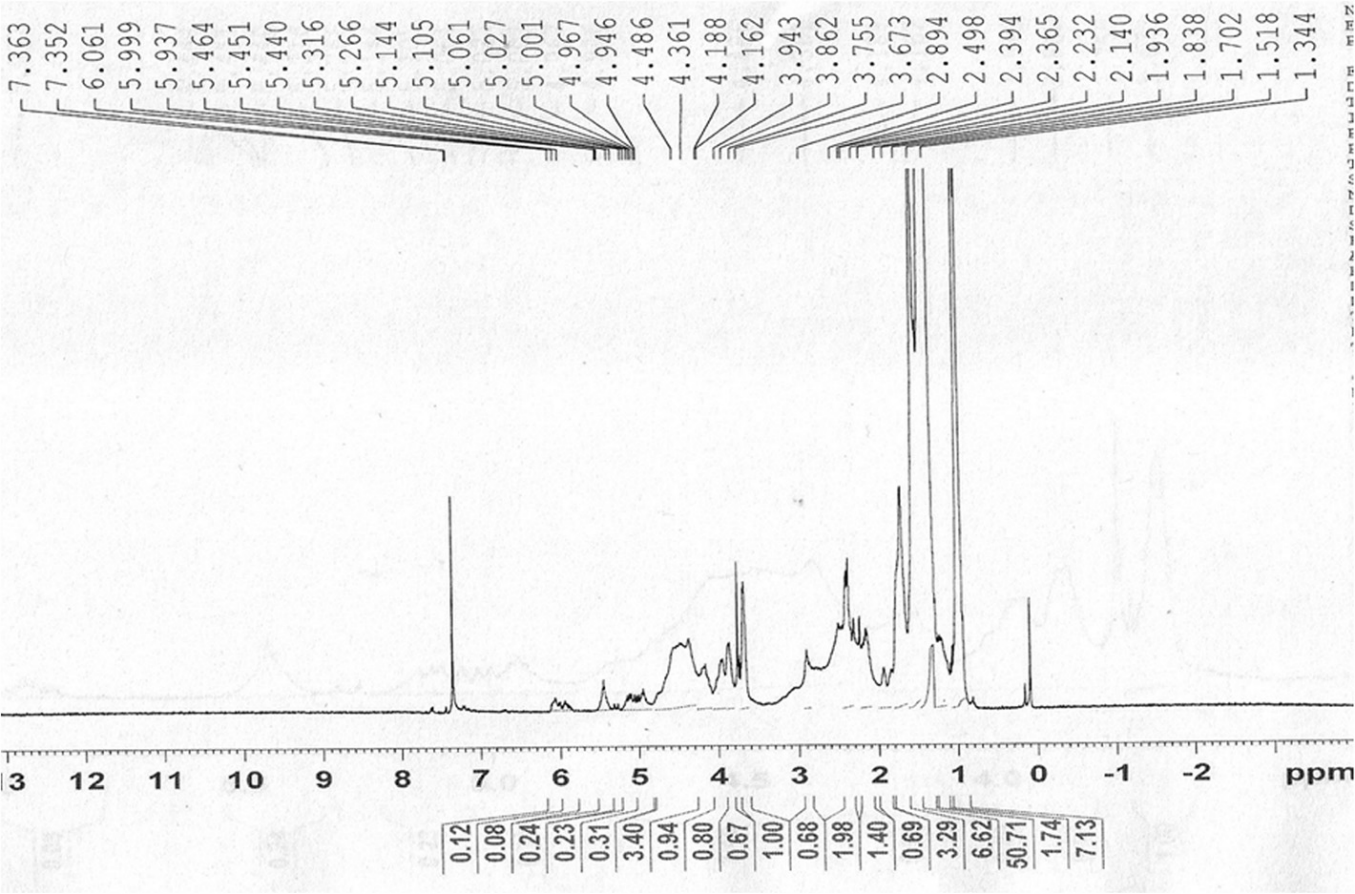
**Proton NMR spectrum of OC, at 400 MHz in CDCl**_**3**_**.**

**Figure 2:**
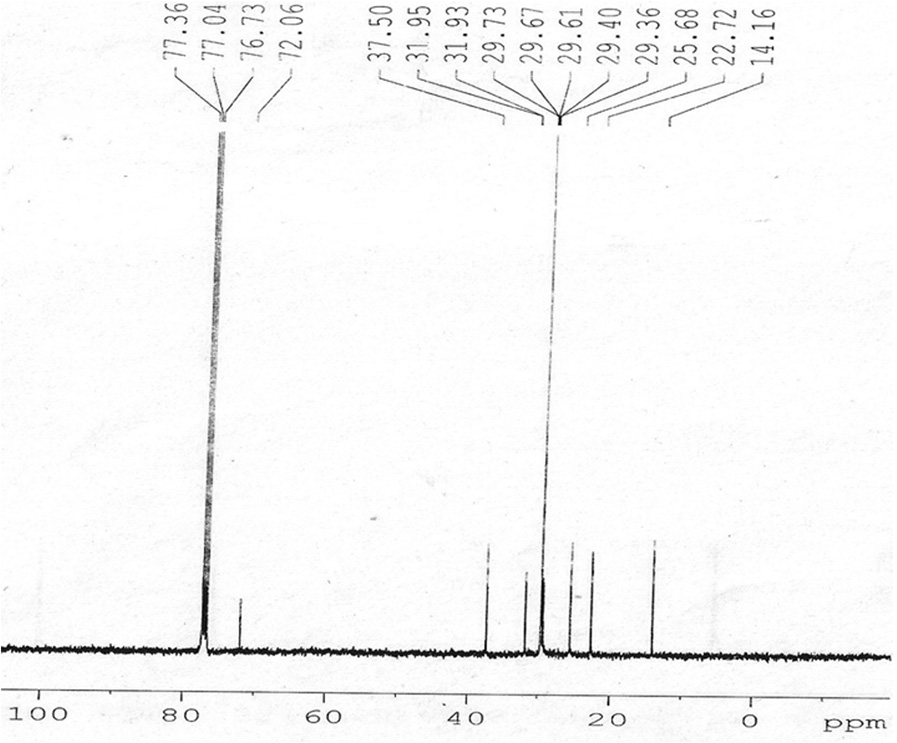
**Carbon NMR spectrum of OC, at 100 MHz in CDCl**_**3**_**.**

**Figure 3:**
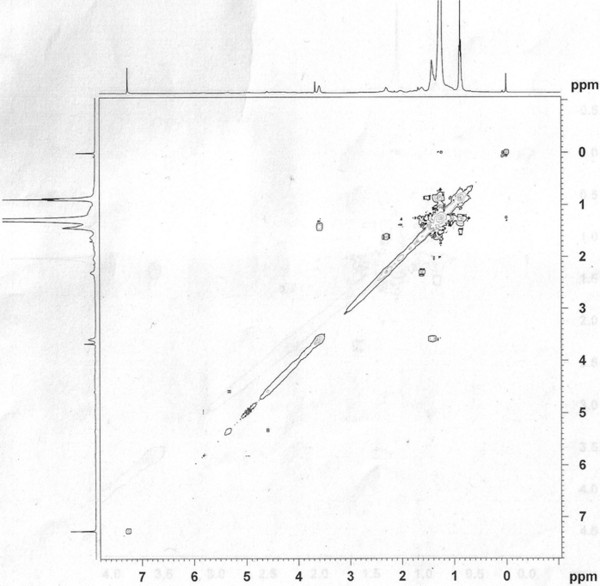
^**1**^**H–**^**1**^**H COSY spectrum of OC at 400 MHz in CDCl**_**3.**_

**Figure 4:**
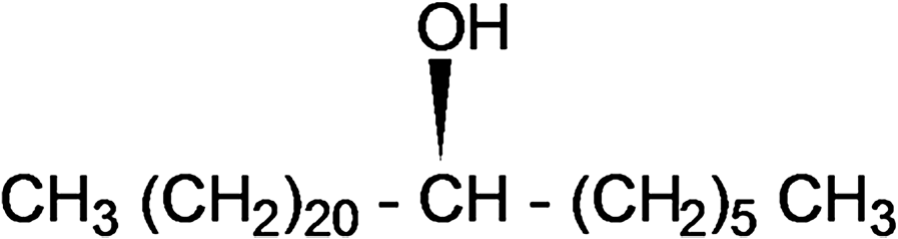
**N-octacosan-7β-ol (OC).**

The compound **OC** designated as octacosyl alcohol was obtained as star-shaped colorless from ratio of petroleum ether-chloroform as eluants. Its IR spectrum exhibited characteristic absorption band for hydroxyl group (3318 cm^-1^) and long aliphatic chain (721 cm^−1^). The mass spectrum of **OC** showed molecular ion peak at *m/z* 411[M+1]^+^ corresponding to a molecular formula of an octacosyl alcohol, C_28_H_59_O. The ion peak arising at *m/z* 325 [CH_3_(CH_2_)_20_CHOH]^+^ indicated the presence of the hydroxyl group at C-7. The ^1^H NMR spectrum of **OC** (Figure 1) showed a one proton broad multiplet at δ 3.67 with half width of 14.1 Hz was ascribed to α-oriented H-7 carbinol proton. The methylene protons resonated between δ 2.36-1.34. Two three-proton triplets at δ 0.98 (*J*= 6.0) and 0.95 (*J*= 6.8) were accounted to C-1 and C-28 primary methyl proton respectively. The ^13^C NMR spectrum of **OC** (Figure 2) displayed signals for carbinol carbon at δ 72.06 (C-7), methylene carbons between 37.50- 22.72 and methyl carbon at δ 14.16 (C-1, C-28). The ^1^H-^1^H COSY spectrum of **OC** (Figure 3) exhibited correlations of Me-1 with H_2_-2 and H_2_-3; H-7 with H_2_-6, H_2_-5, H_2_-8 and H_2_-9; and Me-28 with H_2_-27 and H_2_-26. The absence of any proton singlet beyond δ 3.67 and carbon signal after δ 72.06 in the down field region suggested saturated nature of the molecule. On the basis of foregoing account the structure of **OC** was elucidated as n-octacosan-7β-ol (Figure 4).

### Antibacterial and antifungal study

The values (mm) of inhibitory zones for the bacterial and fungal strain are studied by disc diffusion method and results are mentioned in Table 1. The diameter of the zone of inhibition is expressed in millimeter including the disc (6 mm). The **OC** presented antimicrobial activity against the *Staphylococcus epidermidis, Escherichia coli, Candida albicans* and *Aspergillus niger*, showing the biggest inhibition zones. The results of the Minimum inhibitory concentration (MIC), minimum bactericidal concentration (MBC) and minimum fungicidal concentration (MFC) are presented and mentioned in Table 2. The analyzed compound **OC** expressed strong antibacterial and antifungal potential.

**Table 1:** Antibacterial and antifungal activities of OC as inhibition zones (mm)

**Treatment**	**OC**	**Control**	**Streptomycin**	**Amphotericin B**
**Microorganism**				
*Staphylococcus epidermidis* (Gram +)	14	6	24	-
*Bacillus subtilis* (Gram +)	6	6	17	-
*Escherichia coli* (Gram -)	11	6	16.5	-
*Salmonella typhimurium* (Gram -)	6	6	18	-
*Candida albicans*	12	6	-	17
*Aspergillus niger*	11.5	6	-	15.5

(−) no activity, OC (*n*-octacosan-7β-ol), Control (The filter paper discs, 6 mm, without drug were taken as control), Streptomycin (Positive control for bacterial strain) and Amphotericin B (Positive control for fungal strain).

**Table 2:** Minimum inhibitory concentrations (MIC), minimum bactericidal concentrations (MBC) and minimum fungicidal concentrations (MFC) of OC

**Treatment**		**OC (μg/ml)**	**Streptomycin (μg/ml)**	**Amphotericin B (μg/ml)**
**Microorganism**				
*Staphylococcus epidermidis*	MIC	250	62.5	-
	MBC	250	62.5	-
*Escherichia coli*	MIC	250	50	-
	MBC	500	50	-
*Candida albicans*	MIC	500	-	2.07
	MFC	500	-	2.07
*Aspergillus niger*	MIC	250	-	1.75
	MFC	500	-	2.00

(−) no activity, OC (*n*-octacosan-7β-ol), Streptomycin (Positive control for bacterial strain) and Amphotericin B (Positive control for fungal strain).

### *In vitro* antileishmanial activity by growth kinetics assay

**Figure 5:**
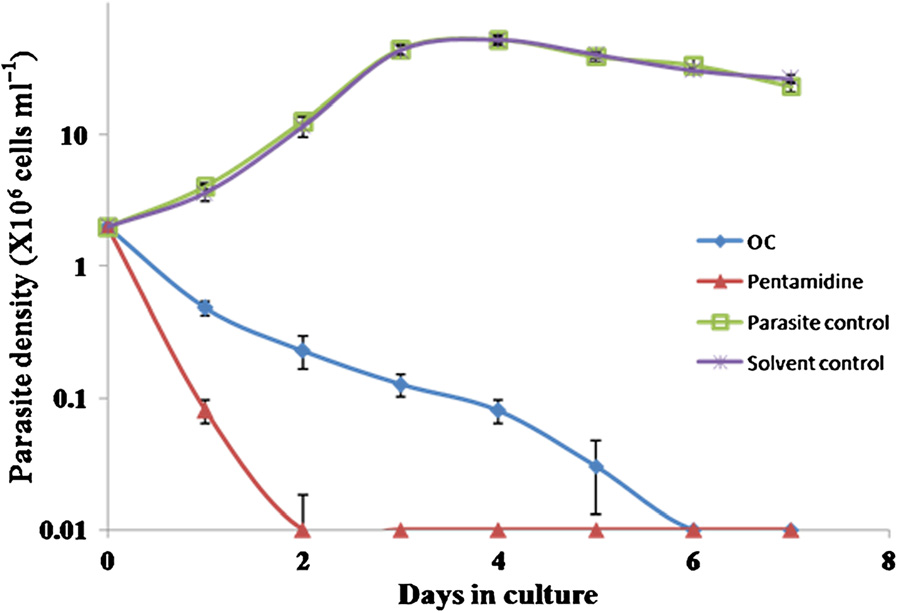
**Analysis of anti-promastigote activity of OC.** Exponential phase promastigotes (2 × 10^6^ cells ml^-1^) of *L. donovani* were incubated with 100 μg ml^-1^ of OC for different time points as described in Methods. Each point corresponds to the mean ± SE of triplicate samples and is representative of one of three independent experiments.

The isolated **OC** was assayed for its cytotoxicity against *L. donovani* promastigotes which exhibited time-dependent killing of promastigotes at a concentration of 100 μg ml^-1^. No viable parasites were observed after 2 and 3 days of incubation with pentamidine and **OC**, respectively (Figure 5). Untreated parasites proliferated at a normal rate.

### Alteration of cellular morphology

**Figure 6:**
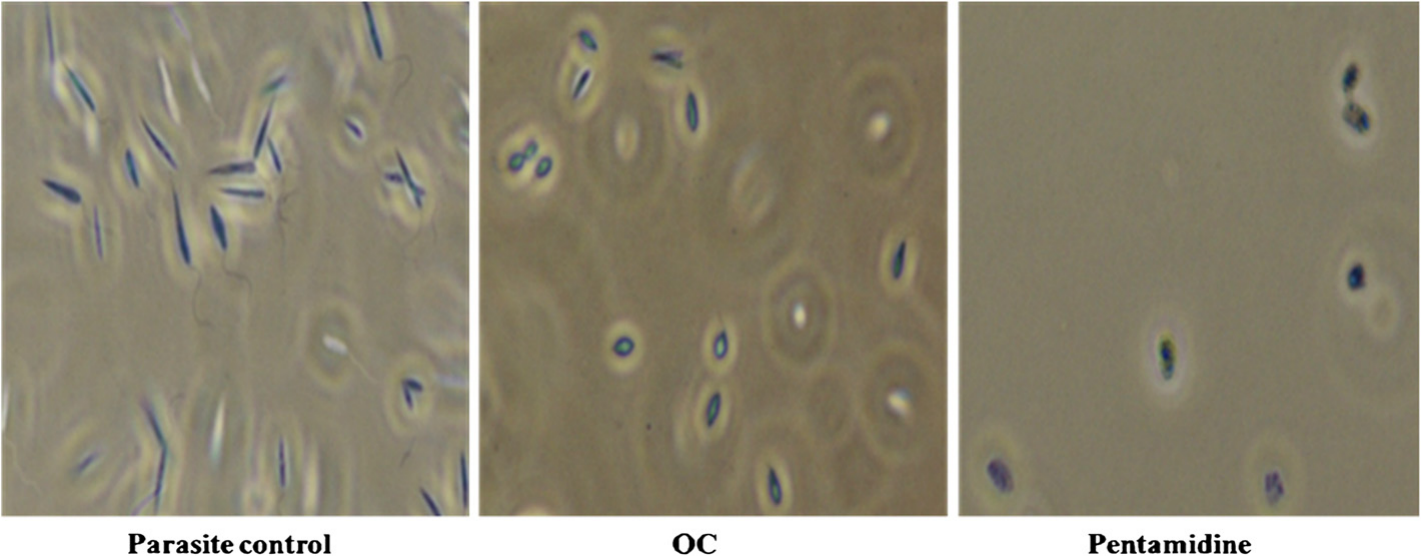
**Analysis of cellular morphology of OC treated promastigotes.** Exponential phase promastigotes (2 × 10^6^ cells ml^-1^) were incubated with 100 μg ml^-1^OC for 96 h and analyzed by light microscopy (400X) as described in Methods.

Visual inspection by phase-contrast microscopy revealed the onset of cell shrinkage and cytoplasmic condensation upon treatment of promastigotes with **OC** (100 μg ml^-1^) (Figure 6), marked by complete circularization of almost all the cells at 96 h post-treatment. Similar morphological changes were observed in pentamidine-treated parasites. Microscopic study indicated that the effect was leishmanicidal rather than leishmanistatic.

### Growth reversibility assay

**Figure 7:**
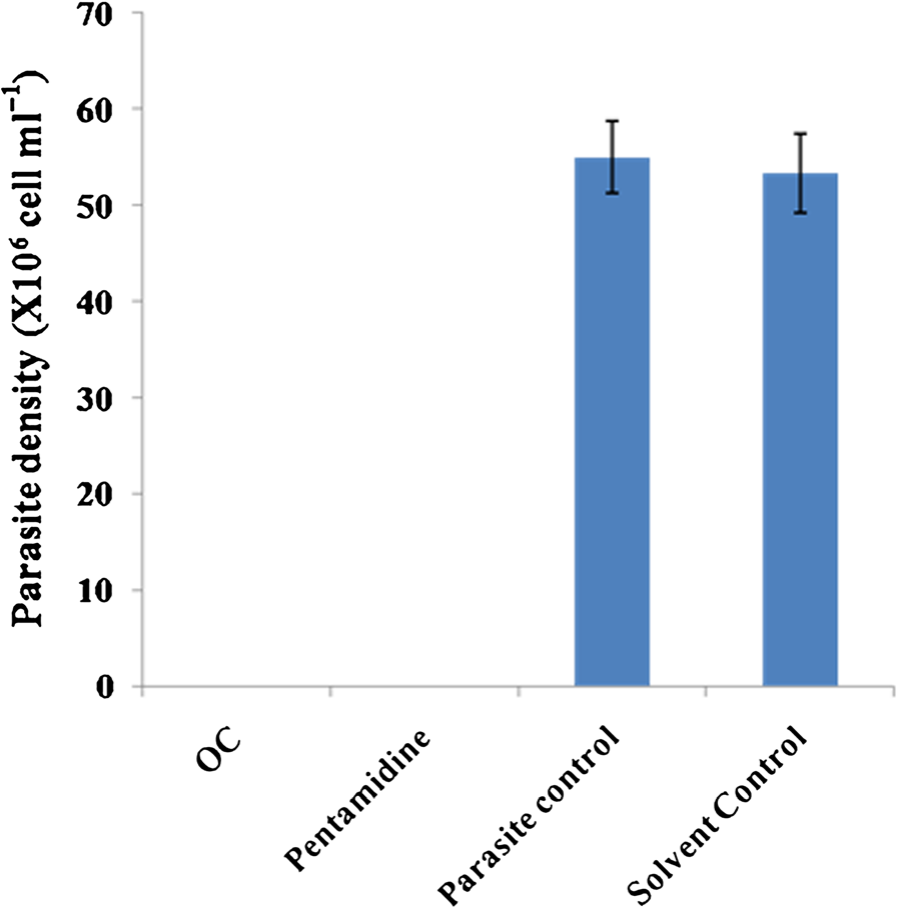
Reversibility analysis of treated promastigotes revealed no reversion of growth in OC and pentamidine treated samples.

To confirm the lethal effect of **OC** for promastigotes, treated and untreated parasites (from the growth kinetics study) were washed and resuspended in fresh medium and their viability was ascertained microscopically after 96 h of incubation. No viable parasites were detected after incubation with **OC**, as it was also observed with pentamidine, confirming their leishmanicidal effect (Figure 7).

### Evaluation of the GI_50_ against promastigotes

**Figure 8:**
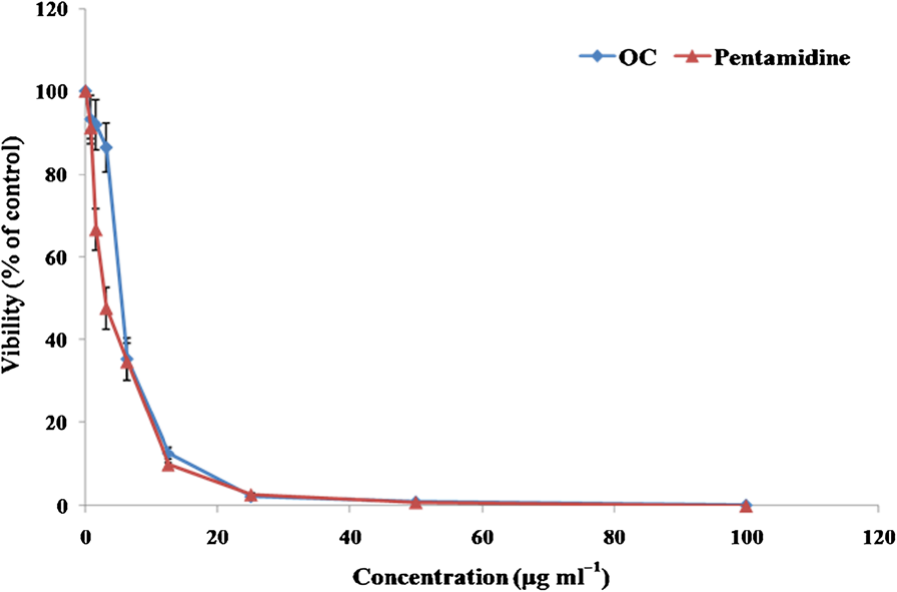
**Estimation of GI**_**50 **_**of OC against promastigotes.** Parasites (2 × 10^6^ cells ml^-1^) were incubated with serial three-fold dilutions of OC (starting at 100 μg ml^-1^) for 96 h and viability was determined microscopically as described in Methods. Each point corresponds to the mean ± SE of triplicate samples and data is from one of three experiments.

The viability of promastigotes after treatment with **OC** was evaluated using a modified GI_50_ assay and its demonstrated dose-dependent inhibition of parasite growth with GI_50_ achieved at 5.35 μg ml^-1^. The established antileishmanial drug pentamidine, used as a positive control, showed a similar trend in dose-dependent parasite killing, with a GI_50_ of 2.9 μg ml^-1^ (Figure 8). Exposure to DMSO (0.2%), representing the highest concentration of diluent, showed no loss of parasite viability.

### Cytotoxicity on mammalian macrophages

**Figure 9:**
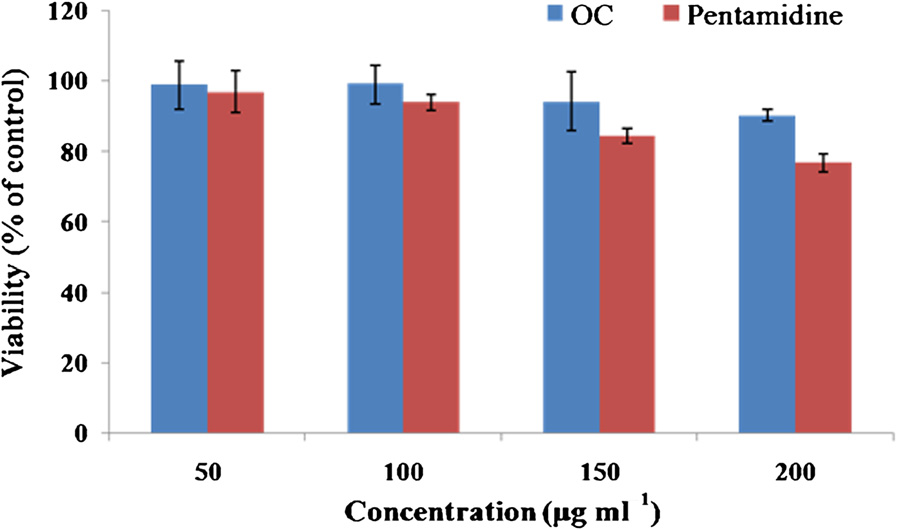
**Determination of adverse toxicity of OC on mammalian macrophages.** Macrophages from peritoneal cavity of mice were incubated for 72 h at 37°C in CO_2_ incubator with increasing concentrations (50, 100, 150 and 200 μg ml^-1^) of OC or pentamidine and viability ascertained. Each bar represents mean ± SE of triplicate samples and data is representative of one of three independent experiments.

Peritoneal macrophages were isolated from mice to check for any adverse side effects of the bioactive **OC**, using pentamidine as the reference drug. The cytotoxicity assay revealed that there was no adverse toxicity of **OC**, even at 200 μg ml^-1^, on mammalian macrophages (Figure 9).

## Discussion

Natural products compounds have been the mainstay of several drug discoveries since the early days of the antibiotic era due to challenge posed by antibiotic resistance. Discovery and development of a new leads of antimicrobial drugs as well as many others could be better explained by reverse pharmacology which is an evolutionary process, based on traditional knowledge which facilitates not only to search experimental database for many novel leads but also minimize time, money and toxicity since several years. VL is considered as an opportunistic infection among immunocompromised patients and mainly treated with toxic pentavalent antimonials, second line drugs such as amphotericin B and pentamidine. Currently there is no vaccine and resistance to antimonial chemotherapy, coupled with its high cost, toxicity and parenteral route of administration, is a matter of great concern in endemic regions of developing countries [27]. Due to severe adverse and toxic profile of synthetic molecules, it is essential requirement for routine screening for safer bioactive compounds from medicinal, aromatic and food plants. Hence there could be no doubt to search for novel antimicrobial agent having antileishmanial potency is one of the core challenges in the current drug discovery programme and this worldwide problem. Previously reports exhibited as anthelmintic [15] and nematocidal activity from extract of *F. parviflora*[17] but still no report of such activity by novel isolated molecule as n-octacosan-7β-ol, **OC** (0.471% yield) may be a new hope for this widespread problem.

## Conclusion

**Figure 10:**
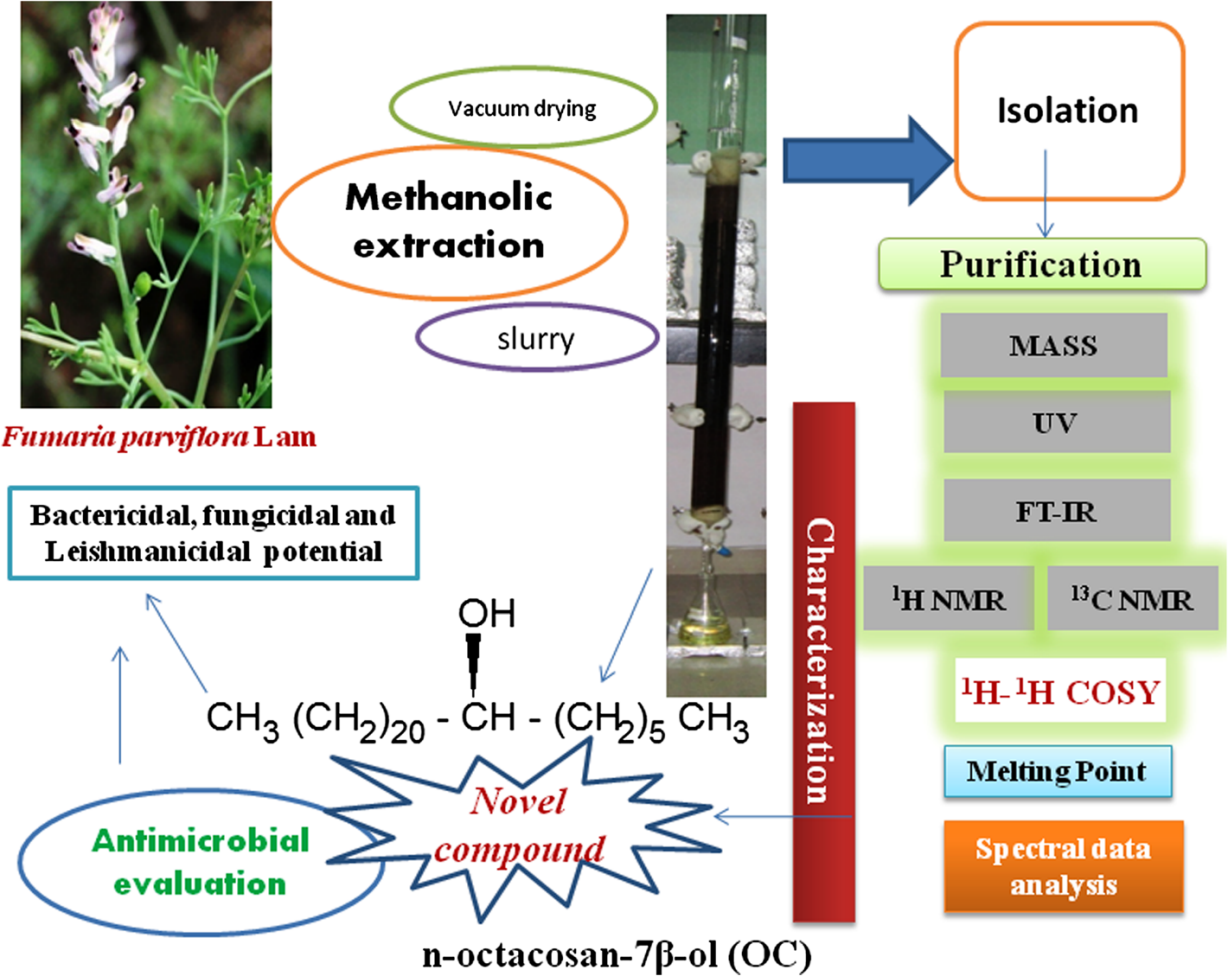
Overview of isolation and characterization of (OC) and its antimicrobial evolution.

In this study we found that **OC** isolated from *F. parviflora* having bactericidal, fungicidal and leishmanicidal activity (Figure 10). In view of the ongoing challenge to introduce new antimicrobial agent as well as potent leishmanicidal drugs which showed dose dependent as well as time dependent parasite killing rate, indicated the potency of **OC** with respect to pentamidine are anticipated to provide a fresh platform. This study showed that **OC** have potent antibacterial and antifungal activities against *S. epidermidis, E. coli, C. albicans* and *A. niger.* These results indicate that the compound might be a practical application in the prevention and protection against bacterial (gram + and gram-), fungal and leishmanial infections in animals and humans.

## Abbreviations

OC: *n*-octacosan-7β-ol; VL: Visceral leishmaniasis.

## Competing interests

The authors declare that they have no competing interests.

## Authors’ contributions

MJ carried out procurement, extraction, study, drafting and isolation work along with AA, MA has made study design, supervise, interpretation of the isolated compound and revision of the manuscript. MI and FA have contributed in Antileishmanial study. All authors read and approved the final manuscript.

## Pre-publication history

The pre-publication history for this paper can be accessed here:

http://www.biomedcentral.com/1472-6882/14/98/prepub
